# Complete chloroplast genome of the genus *Cymbidium*: lights into the species identification, phylogenetic implications and population genetic analyses

**DOI:** 10.1186/1471-2148-13-84

**Published:** 2013-04-18

**Authors:** Jun-Bo Yang, Min Tang, Hong-Tao Li, Zhi-Rong Zhang, De-Zhu Li

**Affiliations:** 1 Germplasm Bank of Wild Species, Kunming Institute of Botany, Chinese Academy of Sciences, Kunming, Yunnan, 650201, China; 2 College of Landscape and Horticulture, Yunnan Agricultural University, Kunming, Yunnan, 650201, China

**Keywords:** Chloroplast genome, Phylogenomics, Species identification, Organelle-scale barcodes, Phylogeny, Divergence hotspot

## Abstract

**Background:**

*Cymbidium* orchids, including some 50 species, are the famous flowers, and they possess high commercial value in the floricultural industry. Furthermore, the values of different orchids are great differences. However, species identification is very difficult. To a certain degree, chloroplast DNA sequence data are a versatile tool for species identification and phylogenetic implications in plants. Different chloroplast loci have been utilized for evaluating phylogenetic relationships at each classification level among plant species, including at the interspecies and intraspecies levels. However, there is no evidence that a short sequence can distinguish all plant species from each other in order to infer phylogenetic relationships. Molecular markers derived from the complete chloroplast genome can provide effective tools for species identification and phylogenetic resolution.

**Results:**

The complete nucleotide sequences of eight individuals from a total of five *Cymbidium* species’ chloroplast (cp) genomes were determined using Illumina sequencing technology of the total DNA via a combination of *de novo* and reference-guided assembly. The length of the *Cymbidium* cp genome is about 155 kb. The cp genomes contain 123 unique genes, and the IR regions contain 24 duplicates. Although the genomes, including genome structure, gene order and orientation, are similar to those of other orchids, they are not evolutionarily conservative. The cp genome of *Cymbidium* evolved moderately with more than 3% sequence divergence, which could provide enough information for phylogeny. Rapidly evolving chloroplast genome regions were identified and 11 new divergence hotspot regions were disclosed for further phylogenetic study and species identification in Orchidaceae.

**Conclusions:**

Phylogenomic analyses were conducted using 10 complete chloroplast genomes from seven orchid species. These data accurately identified the individuals and established the phylogenetic relationships between the species. The results reveal that phylogenomics based on organelle genome sequencing lights the species identification—organelle-scale “barcodes”, and is also an effective approach for studying whole populations and phylogenetic characteristics of *Cymbidium*.

## Background

*Cymbidium* orchids are the best known and most widely grown of all orchids. The genus *Cymbidium* of the orchid family Orchidaceae, consisting of 52–55 species and divided into three subgenera (*Cymbidium*, *Cyperorchis* and *Jensoa*) [1,2], is one of the most well-known and desirable orchids in worldwide horticulture because of its aesthetic appeal and ideal characteristics as a house plant. It is distributed in tropical and subtropical Asia and northern Australia, and species diversity centers are located in NE India, SW China, Indo-China and Malay Archipelago [1-4]. *Cymbidium* orchids were among the earliest to be cultivated, especially in China. Although the *Cymbidium* orchid species is not all widely cultivated, hybrids of *Cymbidium* orchids lend themselves to cultivation. Some commercially important hybrids have been produced for over one hundred years. They make excellent pot plants and cut flowers, which are the most important and popular orchids in commerce.

Because of their ornamental and commercial value, *Cymbidium* orchids have been the subject of taxonomic studies and, particularly, species identification [5-7]. However, so far, there are no efficient methods for identifying the species and cultivars of *Cymbidium*. Traditionally, the taxonomy, species and cultivars identification of the genus *Cymbidium* is based on the morphological traits. However, the assessment of those traits is very difficult, and morphology is affected by environmental factors. Consequently, some species are very difficult to distinguish, and the positions of many species in the evolution and taxonomy of *Cymbidium* are difficult to identify. Owning to widespread artificial hybridization, accurate identification of cultivated varieties via morphological traits is very difficult.

Molecular methods, such as molecular marker techniques, molecular phylogenetics and DNA barcoding, provide effective information for taxonomy, species identification and phylogenetics. In the past decades, the applications of diverse molecular techniques have gained increasing importance in resolving taxonomy, species identification and phylogenetic questions. Choi *et al*. [8] used RAPD markers to investigate the relationships of *Cymbidium*. Obara-Okeyo and Kako [9] identified *Cymbidium* cultivars using RAPD markers. Wang *et al*. [10] reported the cultivar identification in *C. ensifolium* using ISSR markers. Van den Berg *et al*. [5] used ITS and *mat*K to elaborate the phylogenetic relationships of *Cymbidium*. Sharma *et al*. [6] assessed the phylogenetic inter-relationships of *Cymbidium* using ITS. Most of these studies revealed that a limited number of DNA sequences led to relatively little genetic variation within genus *Cymbidium* and therefore phylogenetic resolution and species identification were very difficult.

Owing to the DNA sequencing costs, species identification and molecular phylogenetic analyses were typically limited. It forced investigators to choose a limited number of DNA sequences with a small number of informative loci. In recent years, DNA sequencing costs have fallen dramatically with the rapid development of next-generation DNA sequencing technologies [11-16]. Simultaneously, genomics research rapidly developed allowing the efficient sequencing of large numbers of entire organellar genomes and nuclear genomes. This brought the benefits of affordable genome-scale data collection to phylogenetic resolution and species identification. As a result, it greatly increased phylogenetic resolution and species identification, especially in low taxonomic levels, i.e. genera, species, and populations.

Plastids are one of the essential organelles in plant cells. Molecular differences, based on the dissimilarities in complete chloroplast genome between plant species and individuals, offer a promising means of differentiation. The cp genomes in vascular plants have conserved quadripartite structure, composed of two copies of a large inverted repeat (IR) and two sections of unique DNA, which are referred to as the “large single copy regions” and “small single copy regions” (LSC and SSC, respectively) [17-20]. There are many advantages to the chloroplast genome in contrast to the nuclear genome such as haploid, maternal inheritance, single structure, gene content and genome structure high conservation [21-23]. Complete cp genome sequences have been widely used for plant identification and phylogenetic studies. Moore *et al*. [24] resolved the relationships among basal angiosperms using plastid genome-scale data. Jansen *et al*. [25] used 64 plastid genomes to resolve relationships between angiosperms. Parks *et al*. [26] used chloroplast genomes to increase phylogenetic resolution at low taxonomic levels. Moore *et al*. [27] used 83 chloroplast genomes to resolve the early diversification of eudicots. Wu *et al*. [22] used chloroplast genomes to evaluate identification and breeding in Oncidiinae. Nock *et al*. [28] discussed the plant identification using complete chloroplast genome. Acting as a single genome, it has become the universal method of providing evolutionary information for plant species identification, taxonomy and phylogenetic analysis.

Here, we present the complete nucleotide sequences of eight *Cymbidium* individuals using Illumina sequencing technology of total DNA. The aim of this study was to evaluate the role of the cp genome in taxonomy, species identification and phylogenetics. A phylogenetic tree including 10 complete cp genomes belonging to seven species was reconstructed. Our analyses of eight *Cymbidium* individuals provided detailed genetic data differentiating different individuals and species. This method demonstrated the utility of using complete chloroplast genome sequence information in species identification, taxonomy and phylogenetic resolution of *Cymbidium*.

## Results and discussions

### Genome assembly and PCR-based validation

Using the Illumina Hiseq 2000 system, eight individuals were sequenced to produce 5,703,656 to 7,009,641 paired-end reads (90 bp in average reads length). After screening these paired-end reads through alignment with reference cp genomes, 101,851 to 101,589 reads were mapped to the reference genomes, reaching over 100× coverage on average over the cp genome. After *de novo* and reference-guided assembly, two complete cp genomes were obtained. The other six cp genomes had four to nine gaps, which were then finished gap closure by PCR-based sequencing.

Four junction regions were validated by using PCR-based sequencing in each cp genome. Simultaneously, in order to overcome the errors of heterogeneous indels from homopolymeric repeats [16,29], we corrected the errors by PCR-based validation. We designed 62 pairs of primers based on the variation regions of alignments to validate these sequences in each cp genome (see Additional file 1: Table S1). The validated sequences from eight individuals amounted to 396,800 bp. At the same time, we had compared these sequences directly to the assembled genomes, observing no nucleotide mismatches or indels. This result validated the accuracy of our genome sequencing and assembly. We obtained complete cp genome sequences ranging from 154,769 bp to 156,904 bp in length.

### Genome features and sequence divergence

**Figure 1:**
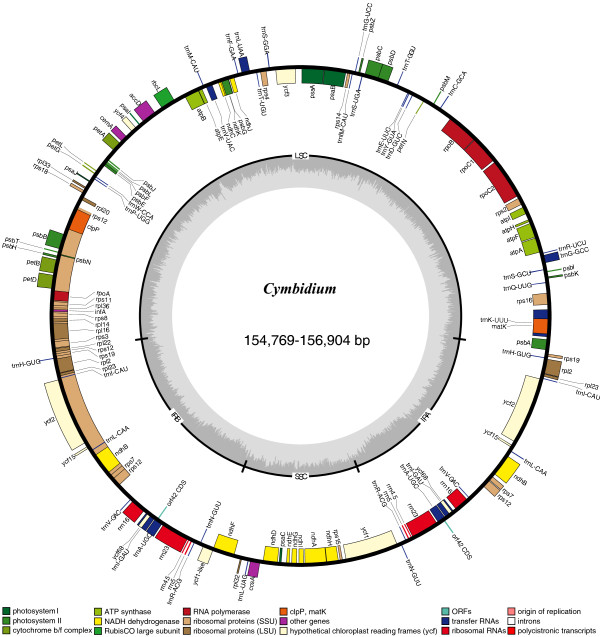
**Gene map of the *Cymbidium *chloroplast genomes.** Genes shown outside the outer circle are transcribed clockwise and those inside are transcribed counterclockwise. Genes belonging to different functional groups are color coded. Dashed area in the inner circle indicates the GC content of the chloroplast genome.

All eight cp genomes were composed of a single the circular double-stranded DNA molecule, and they displayed the typical quadripartite structure of angiosperms, consisting of a pair of IRs (26,321-26,710 bp) separated by the LSC (84,920-85,641 bp) and SSC (16,529-17,929 bp) regions (Figure 1). They encode an identical set of 147 predicted functional genes, of which 123 are unique and 24 are duplicated in the IR regions. The 123 unique genes are comprised of 83 protein-coding, 36 transfer RNA and 4 ribosomal RNA genes, respectively. Sixteen distinct genes, such as *atp*F, *ndh*A, *ndh*B, *pet*B, *pet*D, *rpl*16, *rpl*2, *rpo*C1, *rps*12, *rps*16, *trn*A-UGC, *trn*G-GCC, *trn*I-GAU, *trn*K-UUU, *trn*L-UAA and *trn*V-UAC, contain one intron and two genes (*clp*P and *ycf*3) contain two introns. These introns of all protein-coding genes share the same splicing mechanism as Group II introns [30]. Some exceptional cases were identified in start codons, such as ATC for *ndh*D, ACG for *rpl*2, ACT for *rps*12, GTG for *rps*19 and ATT for *ycf*15. The non-canonical start codons have been detected in other angiosperms [29,31] and tree ferns [32].

Both *ycf*15 and *ycf*68 genes of the IR regions became pseudogenes due to internal stop codons identified in their coding sequences (CDS). A stop codon downstream (153 bp away from the start codon) interrupts the CDS of *ycf*15, whereas the CDS of *ycf*68 is interrupted by two stop codons upstream (57 and 75 bp away from the start codon). Another pseudogene *ycf*1, in the junction region of IRb and SSC exists because of incomplete duplication of the normal copy of ycf1 at the IRa and SSC junction region (Figure 1). Similar mutations occur in the cp genomes of other angiosperm species [29].

In general, *ndh* genes widely exist in higher plants, and eleven subunits of them (*ndh*A-*ndh*K) are encoded in chloroplast genome. Yet there are exceptions: many *ndh* genes are lacking in a number of orchids [22]. In contrast to other orchids previously studies in detail [22,33], having observed all *ndh* gene sequences in *Cymbidium*, we noted that most of them are protein-coding genes except for the genes *ndh*A, *ndh*D, *ndh*F, *ndh*I, *ndh*H, and *ndh*K. These *ndh* genes could have lost their function because of abundance indels generating and stop codons existing in their CDS regions. The loss-of-function of *ndh* genes occurs in many plants including heterotrophic and autotrophic plants [34-43]. As *ndh* genes have important physiological functions, we cannot explain why they lose function or are missing in the cp genome. It is possible that the functional *ndh* genes of the cp genome have been transferred to the nuclear genome [33,41], but this needs to be further explored.

Coding regions occupy 60.16%-60.69% of the cp genomes. 52.46%-53.03%, 1.83%-1.86%, and 5.76%-5.84% of the genome sequence encodes for proteins, tRNAs, and rRNAs, respectively, whereas the remaining 39.31%-39.84% are non-coding regions including introns, intergenic spacers, and pseudogenes. As with other angiosperm cp genomes [31,32], the orchid cp genomes are also AT-rich and the overall AT content ranges from 63% to 63.2%. The genome features of eight cp genomes—paticularly in respect to gene content, gene order, introns, intergenic spacers and AT content—are rather similar.

The cp genomes of the five *Cymbidium* species are distinct from most other monocots in structure and content. Usually, structural rearrangements and gene loss-and gain events are quite common among monocot cp genomes. As a typical example, Poaceae contains three inversions in its LSC regions, which disrupt the canonical order of the cp quadripartite structure and result in the translocation of *rpl*23 from IR to LSC regions [44]. Indels are also common in Poaceae cp genomes such as intron-loss in *rpo*C1 and insertion in *rpo*C2 [45]. Gene-loss (deletion or production of pseudogenes), particularly of the genes *acc*D, *ycf*1, and *ycf*2, are also frequently found in Poaceae cp genomes [46]. In addition, similar events also occur in other monocot families. For instance, *Lemna*, *Dioscorea* and two Acoraceae members, each lost a single gene *inf*A, *rps*16 and *acc*D [45,47,48], respectively, and most *ndh* genes were lost in *Phalaenopsis* and *Oncidium*[22,33]. Beyond that, rearrangements have also been observed in monocots such as the inversion of the SSC region in *Dioscorea*[48]. However, similar to that of standard angiosperm cp genomes, *Cymbidium* cp genomes appeared less rearranged and had very little gene loss-and-gain. Recently, some groups have reported that monocot cp genomes have a similar structure and content to that of the *Cymbidium* species [29,49]. We observed a minor exception—an inversion at the *pet*N and *psb*M regions.

**Figure 2:**
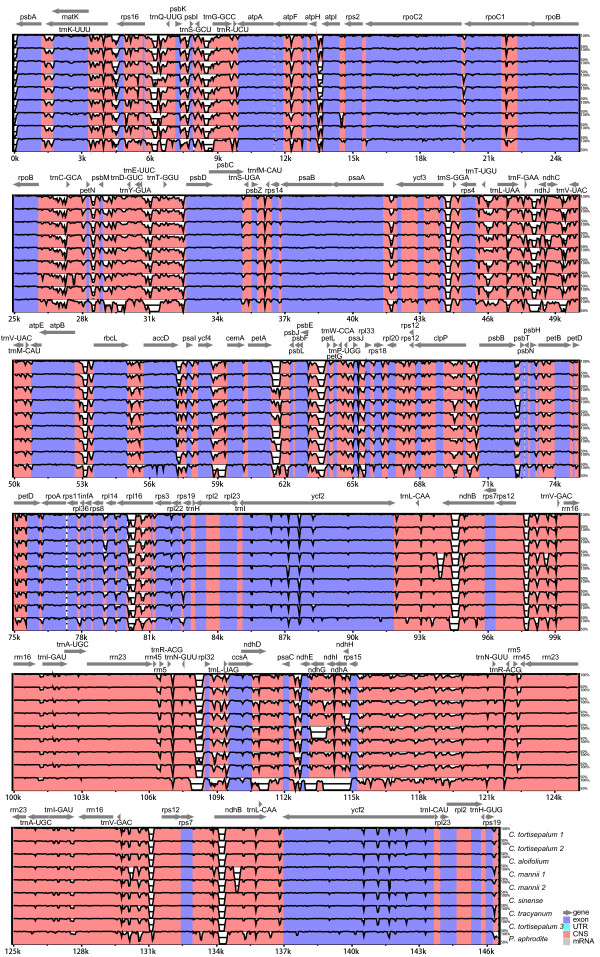
**Visualization of alignment of the 10 Orchidaceae chloroplast genome sequences.** VISTA-based identity plots showing sequence identity between eight sequenced chloroplast genomes and the two published chloroplast genomes of Orchidaceae, with *Oncidium* Gower Ramsey as a reference. Genome regions are color coded as protein coding, rRNA coding, tRNA coding or conserved noncoding sequences.

There is moderate genetic divergence with 3.7% sequence divergence among *Cymbidium* species and individuals. We plotted the sequence identity using mVISTA [50] by aligning the eight cp genomes with a reference, *Oncidium* Gower Ramsey (Figure 2). The whole aligned sequences reveal moderate divergences with more than 30 regions displaying below 60% identity, suggesting that orchid cp genomes have harbored rather large genetic differentiation, especially in noncoding and single copy regions (Figure 2). More than 20 divergent hotspot regions were identified (see Additional file 2: Table S2).

The average genetic divergences of the eight *Cymbidium* individuals were estimated by using *p*-distance. The results showed that the *p*-distance in all individuals, between species and within individuals was 0.009, 0.011 and 0.004, respectively. The results suggested that *Cymbidium* individuals possess moderate genetic divergences both interspecies and intraspecies and that sequence divergences interspecies were significantly more numerous than in intraspecies divergences.

### Repetitive sequences

Four categories of repeats—dispersed, tandem, palindromic and gene similarity repeats—were identified using REPuter [51] and manual verification with copy size 30 bp or longer and sequence identity greater than 90%. Similar to other angiosperm species [52-54], the number of repeats in *Cymbidium* is rather high. In all, 232 repeats were detected in eight *Cymbidium* cp genomes. Numbers and distributions of the four repeat types are similar and conserved among the eight cp genomes. Among these repeats, tandem repeats are the most common accounting for 40% of the total repeats, whereas the gene similarity repeats only occupy 5%. Though a minority of repeats are found in genes such as *inf*A, *rpo*C2, *rps*18 and *rps*3, the majority are located in noncoding regions. The lengths of repeats in *Cymbidium* are much shorter ranging from 30 to 61 bp, whereas much longer repeats such as 91-bp and 132-bp repeats were found in the Poaceae family [53,55]. Previous work suggests that repeat sequences play a role in sequence rearranging and variation production in cp genomes through illegitimate recombination and slipped-strand mispairing [56-58]. Our research also showed that divergent regions of cp the genome were associated with various repeat sequences of genes such as the *rpo*C2 gene. Particularly, the *pet*N-*psb*M gene rearrangement region contained two repeat sequences. The repeat sequences might also be correlated with genome rearrangement in *Cymbidium* cp genomes. Furthermore, these repeats would provide valuable information for developing markers for phylogenetic research and population studies.

### IRs expansion

Gene order in the four junction regions differs among various plant species. The contraction or expansion of the IR regions often results in length variation of the chloroplast genome [18,20,31,33,59]. This study reveals that large expansion occurred in the junction regions of *Cymbidium*. Around the borders of IR-LSC, *Cymbidium* genomes possess the typical monocot cp genome structure, in which the IR regions expand into the *rps*19 gene region. In IRb-LSC, *rpl*22 and its 5’-end adjacent *rps*19 are completely fallen in LSC and IRb, respectively. Similarly, in IRa-LSC, the other copy of *rps*19 in IRa adjoins its 3’-end to *psb*A in LSC. However, the borders of IR-SSC are different from typical monocots, but are similar to certain dicots in that the IR regions expand into the *ycf*1 gene region, that is, the IRb-SSC positions itself between the *ycf*1 pseudogene and *ndh*F. This expansion causes an overlap between the *ycf*1 pseudogene and *ndh*F [20], whereas IRa-SSC resides in the 3’ region of the normal *ycf*1 gene.

Among other monocots, the contraction or expansion of the IR regions were checked. *Lemna*, the basal monocot, had a more contracted IR regions than the basal angiosperm *Amborella*[25,47]. In the borders of IR-SSC, *Cymbidium*, the higher Poaceae and the basal *Lemna* had obvious expansion, whereas little expansion was checked in the other monocots [29,49]. It could suggest that the expansion or contraction may not be associated with phylogenetic relationship among monocots [29].

### Molecular marker identification

The sequence divergence hotspot regions (>200 bp) were identified by whole cp genome-wide comparative analysis. To check the regions, which could be suitable for phylogenetic study, all of the regions were extracted from eight *Cymbidium* cp genomes to be used for phylogenetic analysis using the MP method. The results showed that 32 divergence hotspot regions could be subjected to Orchidaceae phylogenetic analysis. All divergence hotspot regions had a 2% composition of parsimony-informative characters. Interestingly, 11 intergenic regions (*cem*A-*pet*A, *clp*P-*psb*B, *ndh*F-*rpl*32, *pet*A-*psb*J, *psb*A-*trn*K, *trn*L-*ccs*A, *rpl*32-*trn*L, *trn*E-*trn*T, *trn*K-*rps*16, *trn*P-*psa*J, *trn*T-*trn*L) along with the commonly phylogenetic region (*trn*H-*psb*A) had over 3% composition of parsimony-informative characters. Compared with previous studies [5,7,60], all 11 regions harboring high phylogenetic information are newly identified in our current study.

In general, the molecular phylogenetic tree should be congruent with the evolution and life history of a species [61]. Consequently, the MP phylogenetic trees of 11 new divergence hotspot regions were constructed and were evaluated with the partition homogeneity test [62,63]. The results revealed that gene trees of nine new divergence hotspot regions were congruent with the combined species trees of the seven orchid species whole cp genomes (see Additional file 3: Figure S1).

In this study, we disclosed 11 new DNA variable regions harboring highly phylogenetic information, which would be potential molecular markers (see Additional file 4: Table S3) for phylogenetic study and species identification. As a result, it would be helpful if these regions were developed as markers using universal primers in order to reveal the molecular phylogeny of Orchidaceae species and species identification.

### Phylogenomic analyses

Six data partitions (complete cp DNA sequences, protein coding exons, the large single copy region, the small single copy region, inverted repeat region and the introns and spacers) from 10 orchid cp genomes were used to perform phylogenetic analyses. Sequence characteristics for the six datasets are shown in Additional file 5: Table S4. The small single copy region possessed the highest percentage of potential parsimony-informative characters with 3.5%, and the introns and spacers with 3.2%, followed by the small single copy region. The large single copy region and inverted repeat region also contained moderate genetic variation of 2.7% and 0.9% of potential parsimony-informative characters, respectively. Furthermore, the only gene rearrangement region, *pet*N-*psb*M inversion region, is located in the LSC region. Although there were no influences on the phylogenetic tree of *Cymbidium*, the rearrangement region could be considered a homologous character and evolutionary marker of the genus *Cymbidium* that distinguishes the genus *Cymbidium* from other orchid genera. The protein coding exons were highly conserved and had fewer potential parsimony-informative characters less than 1.7%.

**Figure 3:**
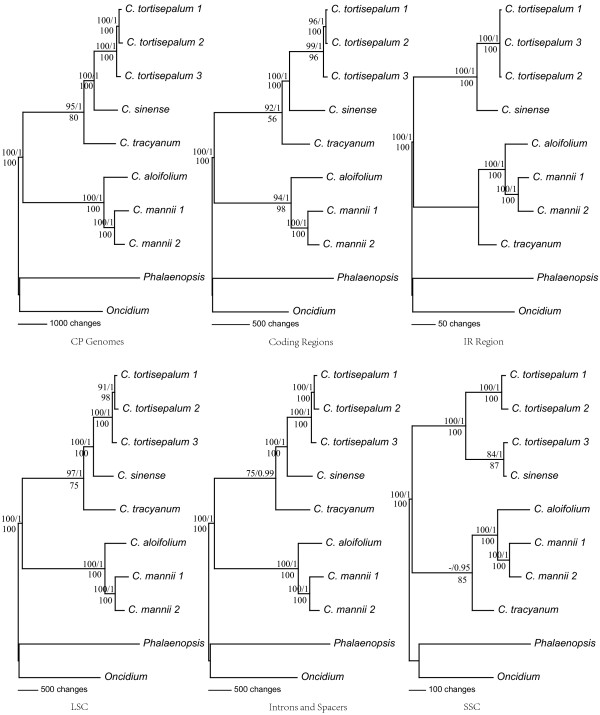
**Maximum parsimony (MP) trees of all the six chloroplast datasets for 10 Orchidaceae individuals.** Numbers above the lines on the left indicate the maximum parsimony (MP) bootstrap of each clade >50%, numbers above the lines on the right indicate the Bayesian posterior probabilities, numbers below each branch are the maximum likelihood (ML) bootstrap of each clade >50%.

Phylogenetic trees with bootstrap values (BS) and posterior probabilities (PP) were built based on the six datasets partitions (Figure 3). The method of data analyses (ML, MP and BA) had no effect on the phylogenetic trees, and the resulting topologies were highly similar in each dataset. Phylogenetic trees of the six datasets partitions were largely congruent with each other, and only the subtle differences of topologies occurred in intraspecial clades. This suggests that there is no conflict between the partitions of cp genome. The results also revealed that the phylogenetic resolution and the support values of nodes increased significantly along with the increase of the sequence (Figure 3).

Compared with the traditional taxonomy of *Cymbidium*, the phylogenomic analyses also revealed that *Cymbidium* consists of three subgenera. However, in view of the percentage of species sampling, this needs to be verified. More importantly, different individuals of the same species from different distributions also generate moderate differentiation and can be clearly distinguished. For example, the three individuals of *C. tortisepalum* make a reliable monophyletic node, while individuals of adjacent distributions maintain sister group relationships. Additionally, the remote distribution individual maintains much divergence from other two individuals (Figure 3). Among the three individuals of *C. tortisepalum* and between the two individuals of *C. mannii*, there are more than 120 and 100 variable sites, respectively, which would provide enough information for cultivars identification and population-level studies. The majority of these variable sites exist in spacer regions such as *rpl*32-*trn*L, *trn*E-*trn*T, *trn*H-*psb*A, *trn*K-*rps*16 and *trn*T-*trn*L. The results showed that whole cp genomes analyses could contribute to species identification, especially in cultivars identification and population-level studies. Cultivars identification plays an important role in the commercial development of orchids. Phylogenomic analyses based on whole cp genomes will light the way on cultivars identification of *Cymbidium*.

Compared with previous short sequence analyses in *Cymbidium*, our cp genomes data contained more than 100 times more parsimony-informative characters, resulting in higher-resolution nodes with much higher support values. Consequently, phylogenomic analyses based on whole cp genomes could overcome defects due to insufficient DNA sequences sampling. Our results suggest that whole cp genomes sequencing would be a feasible, reasonable and effective way for improving resolution of phylogenies, species identification and population-based studies in most land plants. Of course, considering the costs, it could reasonable be asked whether it is worth sequencing whole cp genomes for increasing species identification and phylogenetic relationship resolution. However, that is no longer an issue as a result of the rapid development of the next generation DNA sequencing technologies resulting in the sequencing costs dramatically fallen. Phylogenomics would rapidly develop based on whole cp genome analyses in the near future. However, for the rapidly radiating lineages, the whole cp genomes analyses remain insufficient to fully resolve phylogenetic relationships [26,64,65] and a combined nuclear and organelle genome approach would be feasible.

## Conclusions

Organelle genome sequencing is becoming a valuable way for improving resolution in phylogenetic studies with the rapid development of biotechnological sequencers, especially at low taxonomic levels. Based on the estimates of Cronn et al. [15] and the current development situation, thousands of organelle genomes could be sequenced, which would greatly mitigate current reliance on relatively short sequences in phylogenetic research [26]. It also could promote population genetic studies and species identification. Whole cp genome sequences would provide more integrated and adequate information for phylogenetic and population-based studies, improving efficient discrimination during species identification. In fact, phylogenomic studies have enjoyed recent popularity and the possibility of organelle-scale “barcodes” and population-based studies has been both considered and applied [26,28,66].

Here we sequenced eight orchid individuals involving five *Cymbidium* species using Illumina sequencing-by-synthesis technology. These sequenced cp genomes provided genetic information on the phylogenetics, species identification and population genetic studies for these economically important orchids. These cp genomes contained moderate variations that could provide sufficient phylogenetic information for resolving evolutionary relationships. At the same time, they could also provide adequate genetic information for species identification and population genetic studies. In this study, the cp genomes accurately identified every individual and established the phylogenetic relationships between the species and individuals. The results reveal that it is an effective approach to increase the efficiency and feasibility of species identification and population-based studies while raising new questions regarding the phylogenetic implications of *Cymbidium* giving the characteristics of the cp genomes.

## Methods

### Plant materials

Five species, representing three subgenera of the genus *Cymbidium*, were sampled. We collected healthy, clean and fresh green leaves from adult plants of *Cymbidium*. The voucher herbarium specimens for the eight sampled orchids were all deposited at the Herbarium of Kunming Institute of Botany of the Chinese Academy of Sciences (KUN) (see Additional file 6: Table S5).

### Chloroplast DNA extraction, sequencing, genome assembly, and PCR-based validation

Total DNA enriched for cpDNA extraction from 100 g fresh leaves was obtained according to the procedure outlined in Zhang *et al*. [17,55]. Purified DNA (5 mg) was fragmented and used to construct short-insert libraries according to the manufacturer’s manual (Illumina). DNA from the different individuals was indexed by tags and pooled together in one lane of Illumina’s Genome Analyzer for sequencing.

Since the raw sequence reads mixed non-cp DNA from the nucleus and mitochondria, we isolated the cp sequence reads from the raw sequence reads based on the known cp genome sequences. The filtered cp sequence reads were used to assemble the cp genomes. First, the filtered short reads were assembled into non-redundant contigs using SOAPdenovo [67], a *de novo* sequence assembly software, with *k* = 31 bp and scaffolding contigs with a minimum size of 100 bp. Then, all contigs were mapped to the reference cp genomes in Orchidaceae [22,33] using BLAST (http://blast.ncbi.nlm.nih.gov/) searches from NCBI with default parameters. Third, the orders of aligned contigs were determined according to the reference genomes. Finally, gaps between the *de novo* contigs were replaced with consensus sequences of raw reads mapped to the reference genomes.

Based on the reference genomes, the four junctions between LSC/IRs and SSC/IRs were confirmed with PCR-based product sequencing, respectively. To avoid assembly errors and to obtain high quality complete cp genome sequences, validation of assembly was also carried out with intensive PCR-based sequencing. We designed 62 pairs of primers based on the variation regions of the eight preliminary cp genome assemblies. PCR products were sequenced using the BigDyeV3.1 Terminator Kit for ABI 3730xl (Life Technologies). Sanger sequences and assembled genomes were aligned using Geneious [68] to determine if there were any differences. The final complete cp genome sequences were deposited into GenBank (see Additional file 6: Table S5).

### Genome annotation and repeat analysis

We performed annotation of the sequenced genomes using DOGMA [69], coupled with manual corrections for start and stop codons and for intron/exon boundaries to match gene predictions from the sequenced cp genomes in Orchidaceae [22,33] of GenBank and the Chloroplast Genome Database (ChloroplastDB) http://chloroplast.cbio.psu.edu/[70]. The sequences of identified tRNA genes were obtained using DOGMA and tRNAscan-SE (version 1.23) [71]. The functional classification of cp genes was referred to CpBase (http://chloroplast.ocean.washington.edu/) and ChloroplastDB. The annotated GenBank files of the cp genomes of *Cymbidium* were used to draw gene maps using OrganellarGenome DRAW tool (OGDRAW) [72].

Both direct and inverted repeats were assessed via REPuter [51]. Four types of repeats—dispersed, tandem, palindromic and gene similarity repeats—were observed within the *Cymbidium* cp genomes. The maximal length of gap size between palindromic repeats was restricted to 3 kb. Overlapping repeats were incorporated into one repeat motif whenever possible. A given region in the genome was defined as only one repeat type, and the tandem repeat was prior to the dispersed repeat if one repeat motif could be identified as both tandem and dispersed repeats.

### Phylogenomic analyses

The eight sequenced *Cymbidium* cp genome sequences and two public orchid cp genomes were aligned using MAFFT version 5 [73] and five loci were adjusted manually according the criteria of reducing gaps. Three ambiguously aligned loci, i.e., ‘N’ were excluded from the analyses. The unambiguously aligned DNA sequences were used for phylogenetic and species identification analyses. To check the utilities of phylogenetic and species identification from different regions, simultaneous analyses were carried out based on the following data: (1) the complete cp DNA sequences; (2) protein coding exons; (3) the large single copy region; (4) the small single copy region; (5) the inverted repeat region; and (6) the introns and spacers. All alignments used in this study were deposited in DRYAD (accession no. doi:http://10.5061/dryad.14214).

Maximum likelihood (ML) and maximum parsimony (MP) analyses were conducted using PAUP 4.0b10 [74,75]. Characters were treated as unordered and unweighted. The best model and parameter settings were chosen according to the Akaike information criterion (AIC) as suggested by Modeltest V3.7 [76,77] for the ML analyses. Heuristic searches were conducted with tree-bisection reconnection (TBR) branch swapping, MulTrees ON, and 10,000 random taxon addition replicates holding 20 trees at each step. Bootstrap support (BS) values for individual clades were calculated by running 1,000 bootstrap replicates of the data, with starting trees acquired by a single replicate of random stepwise addition of taxa under TBR branch swapping, with MulTrees ON. The consistency index (CI), retention index (RI), rescaled consistency index (RC), and pairwise distances were obtained through PAUP 4.0b10 as the actual number of site differences excluded indels.

Bayesian analyses (BA) were conducted using MrBayes 3.2 [78,79]. The best model and parameters settings were chosen according to the Akaike information criterion (AIC) as suggested by ModelTest v3.7 [76,77]. The results were based on the best-fit models of the AIC test. Four independent Markov Chain Monte Carlo chains were run simultaneously and sampled every 100 generations for a total of 1,000,000 generations. To establish the “burn-in” phase, i.e., log probability values stationarity, a plot of generations against log likelihood scores was generated; these burn-in trees were discarded from the analysis.

### *p*-distance calculation

To assess the utility of complete cp genome sequencing in species identification and population genetics, the aligned complete cp genome sequences were used to calculate p-distance with MEGA5 [80]. Missing data were treated as complete deletions.

### Molecular marker identification

To examine the divergence regions for phylogenetic applications, all the regions, including coding regions, introns and intergenic spacers from eight *Cymbidium* cp genomes, were sequentially extracted. Every homologous region was aligned using MUSCLE [81] and further manual adjustments were made where necessary. As a result, the percentage of variable characters for every region was calculated.

For the divergence hotspot regions, the maximum parsimony method was used to construct the phylogenetic trees with PAUP4.0b10 to check the congruence of evolution and life history of the species. Heuristic tree searches were conducted with 10,000 random-taxon-addition replicates holding 20 trees at each step and tree bisection-reconnection (TBR) branch swapping, with the “MulTrees” option in effect. Non-parametric bootstrap analysis was conducted using 1,000 replicates with TBR branch swapping.

## Competing interests

The authors declare that they have no competing interests.

## Authors’ contributions

JBY, HTL and DZL designed research and wrote the paper. JBY, MT, HTL and ZRZ performed research. HTL analyzed data. All authors read and approved the final manuscript.

## Supplementary Material

Additional file 1: Table S1 Primers used for gap closure, assembly and junction verification. Click here for file

Additional file 2: Table S2 Percentage of parsimony-informative characters in 32 divergence hotspot regions among eight *Cymbidium* individuals. Click here for file

Additional file 3: Figure S1 Maximum parsimony (MP) trees of nine regions from the 11 new DNA divergence hotspot regions of 10 Orchidaceae individuals. Click here for file

Additional file 4: Table S3 Primers for 11 potential molecular markers. Click here for file

Additional file 5: Table S4 DNA site variation and tree statistics for the six datasets used in the phylogenomic analyses presented in this study. Click here for file

Additional file 6: Table S5 Sampled species and voucher specimens of *Cymbidium* used in this study. Click here for file
