# Supplementary material for: Complete chloroplast genome of the genus Cymbidium: lights into the species identification, phylogenetic implications and population genetic analyses
Source: BMC Evol Biol. 2013 Apr 18;13:84. doi: 10.1186/1471-2148-13-84 (PMC3644226; doi:10.1186/1471-2148-13-84)
Supplement: Additional file 1: Table S1 — Primers used for gap closure, assembly and junction verification. [file 1471-2148-13-84-S1.doc]

Table S1. Primers used for gap closure, assembly and junction verification.

| Primer | Forward sequence | Reverse sequence |
| --- | --- | --- |
| P01 | 5' ATCCTTGTCATTCTGCCATT 3' | 5' AAGACCACGACTGATCCTG 3' |
| P02 | 5' TCCACCGATGAGTTAGCA 3' | 5' ATGTTGAGGAAGGCAGAAT 3' |
| P03 | 5' TGGCTGAACCGATACATA 3' | 5' GAGATGGATGCGAAAAGAC 3' |
| P04 | 5' TTTACAAAAGCCGACCTG 3' | 5' TTGGATAGAAATCGTGAGA 3' |
| P05 | 5' ATTCCCGCTACCCGCTAT 3' | 5' CCGAAGAGGCTGAAACACT 3' |
| P06 | 5' GTCAAGTCTACCCATTCCC 3' | 5' ACTACCGTTCAGAGTTGGA 3' |
| P07 | 5' AAGGATAAGTACAATGGGAT 3' | 5' TTGCCTATGCTTTTACGG 3' |
| P08 | 5' AAATGAGGAAAGGAATAG 3' | 5' TCGCAAACAGAGTTAGTA 3' |
| P09 | 5' TACTAACTCTGTTTGCGAT 3' | 5' TCTTCTTATGCTTATGGAT 3' |
| P10 | 5' GGTATGAATTGCATCTTGATG 3' | 5' AAGTGGAAAAGCGGGAAA 3' |
| P11 | 5' GTCATCTAAACCCGAAAAGT 3' | 5' ATTTGAAGAGCGACCATACT 3' |
| P12 | 5' ATTCTTTGGATTTGATGG 3' | 5' TACTTTGTTTCGTTTTCG 3' |
| P13 | 5' GGAGGATTACAAACATGA 3' | 5' GGGAAATACCAAAGAAGT 3' |
| P14 | 5' CTCCTCCGTAACGAGACAT 3' | 5' TTTCCTCCTCCTTCATTTT 3' |
| P15 | 5' GAATAAGAAGGGATAGGT 3' | 5' TTAAACTGAGTTCCACTG 3' |
| P16 | 5' TTACTAAAGAGGGAGCCA 3' | 5' AAGGGAAGGAAAGGATGA 3' |
| P17 | 5' AAAGCCGCATAGTTGAGA 3' | 5' GGTTGGAGTCCGTATAGCC 3' |
| P18 | 5' TGGGCTCTTTCATCAACT 3' | 5' CAGGGTCTCCATCACTTC 3' |
| P19 | 5' AGACCTAGATTTGCCTCA 3' | 5' GAGAATACGGCTAAACGAC 3' |
| P20 | 5' CCGCCTATTCTTCTTTAT 3' | 5' GGTAGCATCGTCCTTTGT 3' |
| P21 | 5' GACCGAATCAAATAAAGAAT 3' | 5' CACCAATCCAATCCCAAG 3' |
| P22 | 5' ACGATGAAGATAAGGATGGGAGA 3' | 5' TGCGATTGCTGGAAAGACTA 3' |
| P23 | 5' CAGTTTGTAAAGGTTCAGTTGT 3' | 5' TTTGATGAAGAATCCAATACC 3' |
| P24 | 5' TACCGTCATACCTGGATTG 3' | 5' ATACCTGGGGTTATAGTTG 3' |
| P25 | 5' GATTCTGCTGGATCATTG 3' | 5' GATTTACGAAAAGGTTGTT 3' |
| P26 | 5' CTTTGAACGGAGCCTGGATA 3' | 5' GTGCGACTTGTCAGACAGATTG 3' |
| P27 | 5' CATCTACAAGGAGACAAGT 3' | 5' GTGGAAACTAAGAGGAATA 3' |
| P28 | 5' GGGACTACACCACTTATG 3' | 5' ACATCAAGTTGGCTCATC 3' |
| P29 | 5' ACAAGCGATTGGAGATGA 3' | 5' TTTGGTCCCGAGGTAAGG 3' |
| P30 | 5' TCAAAGTTACATACCACGAC 3' | 5' ATTGGGCACACTACAGAA 3' |
| P31 | 5' TATCGGCAATAATGTCCCTACC 3' | 5' TTCATTAGCACCCGAGGTCTTA 3' |
| P32 | 5' TTTTGGACTTGTTACATAGG 3' | 5' ACTTATTGCTTCGTATTGTC 3' |
| P33 | 5' TTCTTCCCTACGAGTTCC 3' | 5' ATCTACGATGGTCCCTGTT 3' |
| P34 | 5' ATAGGGATTGTAGTTGTCAG 3' | 5' ATACATTCCATACCTTGCT 3' |
| P35 | 5' ATTTGGAATCTGGGCTCTT 3' | 5' GGTTCTCGGACGACTCAC 3' |
| P36 | 5' ACGGTAAACGCTGGGTAG 3' | 5' AGTTCGGGATGGATTGGT 3' |
| P37 | 5' ACAGGATGTGATACGATGAG 3' | 5' AAGGAATAACACGACCGA 3' |
| P38 | 5' GTGGCGGCATAAGGCATA 3' | 5' CCCGAAGAAATCCAACGAA 3' |
| P39 | 5' TTTATTGTAGACACCAGACG 3' | 5' TCTATCGGTATCTATGGACTG 3' |
| P40 | 5' ATTTCTTAGACCAGTTATT 3' | 5' GCAGTCGTTTTAGTATGT 3' |
| P41 | 5' TATTTCGGTATTCTTTAGGTA 3' | 5' ACTTATCTTCCCTGTCCC 3' |
| P42 | 5' AGTTGTTGCTGATACCTCCTT 3' | 5' ATCCCAGTTCCATTCATTTC 3' |
| P43 | 5' CGGAGCCACTACGAAGAAG 3' | 5' CTAACGGTCAAAGCGAGCC 3' |
| P44 | 5' ATGAGACCCGATTATTGAC 3' | 5' CTAAGTAGTAAGCCCACCC 3' |
| P45 | 5' ATTCGTGCTGATTCTTCCC 3' | 5' TTGATTGTTTCGCTCCTGT 3' |
| P46 | 5' GTTTCATTGATTCCTCCTA 3' | 5' ATTCCATTGATACAGAGCC 3' |
| P47 | 5' GGCTGATGGTAAATTCATTGA 3' | 5' GTCGCGAATTGAAGAGTCTAA 3' |
| P48 | 5' AAACAATTTCATGAATCTACT 3' | 5' TTGAATTCCTGATTCTTTATA 3' |
| P49 | 5' AGGTATGAATTGCATCTTGAT 3' | 5' GCTGCTTTAATGGTAGTGTTT 3' |
| P50 | 5' GCAAATGATGTCCCCTATCT 3' | 5' AAAAGGAAAAGTGGAAAAGC 3' |
| P51 | 5' GCCTGCCTGCCATCCACACC 3' | 5' GGTTCAAGCTTTCCCCAGCCCC |
| P52 | 5' AGAACTTGTACCTTGTCAACCGATAAC 3' | 5' GCTGTCAAATCGCCGATGATACCTC |
| P53 | 5' ACAGAACAAAAGAAGATATTCACAGTAGA 3' | 5' TCTTTAGGTCGGTTACCAGTTTCA |
| P54 | 5' AGAAAAAGCACTTCCGAATGGATCT 3' | 5' TTCAGGCGGTCTACGAGCTG 3' |
| P55 | 5' CAGCTCGTAGACCGCCTGAA 3' | 5' TGGTGGGGAGGAGACAATAAATTGGGG 3' |
| P56 | 5' TCGCCCTCTTTTTGCCATTTGACT 3' | 5' CGCAAAGGGGTCTACGAATAATTCTGGG 3' |
| P57 | 5' AGGCAGAATGCCGTCGCCTA 3' | 5' TCTATCAGGAAAAGGGACAGTTAATAGGA 3' |
| P58 | 5' TCCTATTAACTGTCCCTTTTCCTGATAGA 3' | 5' TCCAATGCTTCAATGTTGGGATTAGT 3' |
| P59 | 5' AGCACTTGCTGCAATGGGTCGT 3' | 5' AGTGACTCCTTTTTATCGTTGTATGTGAA 3' |
| P60 | 5' TCGAATTGGAGCCATAAGACTCTATCCA 3' | 5' CGTTGCAATTGATGTTCGATCCCG 3' |
| P61 | 5' CGGGATCGAACATCAATTGCAACG 3' | 5' CGATGTGGTAGAAAGCAACGTGCG 3' |
| P62 | 5' ACCTGACGGTTCACAAACGGCT 3' | 5' ACCTGCGATTCCATAGAAAGACATCACG 3' |
