# Supplementary material for: Complete chloroplast genome of the genus Cymbidium: lights into the species identification, phylogenetic implications and population genetic analyses
Source: BMC Evol Biol. 2013 Apr 18;13:84. doi: 10.1186/1471-2148-13-84 (PMC3644226; doi:10.1186/1471-2148-13-84)
Supplement: Additional file 2: Table S2 — Percentage of parsimony-informative characters in 32 divergence hotspot regions among eight Cymbidium individuals. [file 1471-2148-13-84-S2.doc]

Table S2. Percentage of parsimony-informative characters in 32 divergence hotspot regions among eight *Cymbidium* individuals.

| Divergence hotspot regions | Characters (n) | Informative sites (n) | Informative sites (%) |
| --- | --- | --- | --- |
| *nhd*C-t*rn*V | 745 | 15 | 2.01 |
| *pet*D_intron | 886 | 18 | 2.03 |
| *ycf*4-*cem*A | 735 | 15 | 2.04 |
| *trn*V_intron | 587 | 12 | 2.04 |
| *trn*L_intron | 899 | 19 | 2.11 |
| *rbc*L-*acc*D | 977 | 21 | 2.15 |
| *rps*16-*trn*Q | 1113 | 24 | 2.16 |
| *ndh*A-*ndh*D | 275 | 6 | 2.18 |
| *acc*D-*psa*I | 455 | 10 | 2.20 |
| *trn*Q-*psb*K | 348 | 8 | 2.30 |
| *atp*B-*rbc*L | 1200 | 28 | 2.33 |
| *trn*S-*rps*4 | 299 | 7 | 2.34 |
| *psa*J-*rpl*33 | 640 | 15 | 2.34 |
| *pet*N-*psb*M | 838 | 20 | 2.39 |
| *trn*L-*trn*F | 369 | 9 | 2.44 |
| *rpl*16_intron | 1348 | 33 | 2.45 |
| *rpo*B-*trn*C | 1498 | 38 | 2.54 |
| *psa*C-*ndh*E | 572 | 15 | 2.62 |
| *rps*14-*ycf*1 | 337 | 9 | 2.67 |
| *rps*4-*trn*T | 367 | 10 | 2.72 |
| *psb*A-*trn*K | 260 | 8 | 3.08 |
| *ndh*F-*rpl*32 | 719 | 23 | 3.20 |
| *trn*E-*trn*T | 1311 | 44 | 3.36 |
| *rpl*32-*trn*L | 648 | 22 | 3.40 |
| *cem*A-*pet*A | 247 | 9 | 3.64 |
| *pet*A-*psb*J | 1063 | 41 | 3.86 |
| *trn*P-*psa*J | 381 | 15 | 3.94 |
| *rps*19-*psb*A | 388 | 16 | 4.12 |
| *rpl*32-*ccs*A | 208 | 10 | 4.81 |
| *trn*K-*rps*16 | 609 | 32 | 5.25 |
| *clp*P-*psb*B | 630 | 43 | 6.83 |
| *trn*T-*trn*L | 647 | 52 | 8.04 |
