# Supplementary figures and images for: Complete chloroplast genome of the genus Cymbidium: lights into the species identification, phylogenetic implications and population genetic analyses
Source: BMC Evol Biol. 2013 Apr 18;13:84. doi: 10.1186/1471-2148-13-84 (PMC3644226; doi:10.1186/1471-2148-13-84)

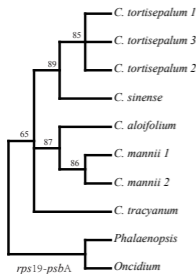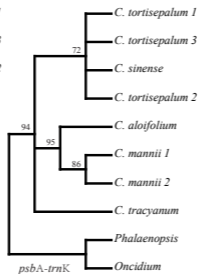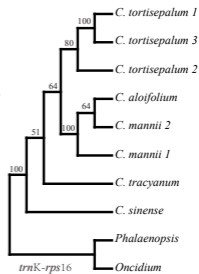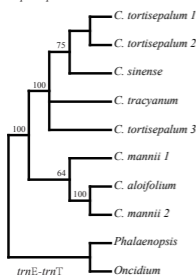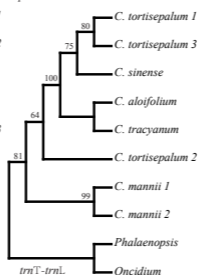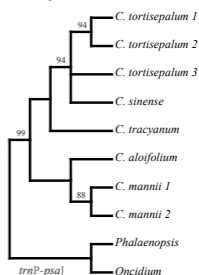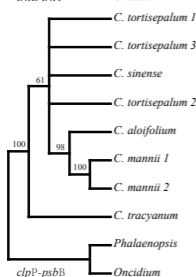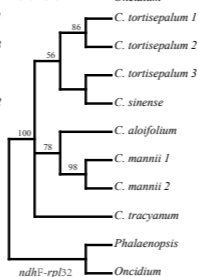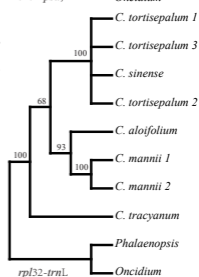

Supplement: Additional file 3: Figure S1 — Maximum parsimony (MP) trees of nine regions from the 11 new DNA divergence hotspot regions of 10 Orchidaceae individuals. [file 1471-2148-13-84-S3.pdf]
