# Supplementary material for: Complete chloroplast genome of the genus Cymbidium: lights into the species identification, phylogenetic implications and population genetic analyses
Source: BMC Evol Biol. 2013 Apr 18;13:84. doi: 10.1186/1471-2148-13-84 (PMC3644226; doi:10.1186/1471-2148-13-84)
Supplement: Additional file 4: Table S3 — Primers for 11 potential molecular markers. [file 1471-2148-13-84-S4.doc]

Table S3. Primers for 11 potential molecular markers.

| Marker | Forward primer | Reverse primer |
| --- | --- | --- |
| *cem*A-*pet*A | 5'TCACTCAACCCATGGTTGGGAACT3' | 5'TCGCCCAGTTGCTTCTCGTGG3' |
| *clp*P-*psb*B | 5'GCATCTTCCTCTCCAGGACTCCGA3' | 5'CGAGCCGGCCCAACCAGAAA3' |
| *ndh*F-*rpl*32 | 5'AGGAAAGCCCACATACGACGA3' | 5'TGACTTTCCGGTGGAAATAGATTTAGC3' |
| *pet*A-*psb*J | 5'TCTTGGCATCTGTGATTTTGGCACA3' | 5'ACGGATCCTATTCCGGGTTGGGT3' |
| *psb*A-*trn*K | 5'CCAGTTGCAGAAGCGACCCCA3' | 5'TGGACATTATCCGTATCAATGATCTGGTGG3' |
| *rpl*32-*trn*L | 5'GCTAAATCTATTTCCACCGGAAAGTCA3' | 5'GCCGCCACTCGGACTCGAAC3' |
| *trn*E-*trn*T | 5'TCGAATCCCCGTTGCCTCCT3' | 5'TGGCGTCACTCTACCACTGAGTT3' |
| *trn*K-*rps*16 | 5'ACCTTTTCCCACATCAGGCACT3' | 5'AGGTGTTCAACCCACAGGAACTG3' |
| *trn*L-*ccs*A | 5'GTTCGAGTCCGAGTGGCGGC3' | 5'GGAAAATGTCCCGAATAAATCCAACG3' |
| *trn*P-*psa*J | 5'CGCGCTACCAAGCTGCGCTA3' | 5'AGCACAGGTGCCGTTGAGAGA3' |
| *trn*T-*trn*L | 5'AGCCGGCTATCGGAATCGAACC3' | 5'CCGTAGCGTCTACCAATTTCGCCA3' |
