# Supplementary material for: Complete chloroplast genome of the genus Cymbidium: lights into the species identification, phylogenetic implications and population genetic analyses
Source: BMC Evol Biol. 2013 Apr 18;13:84. doi: 10.1186/1471-2148-13-84 (PMC3644226; doi:10.1186/1471-2148-13-84)
Supplement: Additional file 5: Table S4 — DNA site variation and tree statistics for the six datasets used in the phylogenomic analyses presented in this study. [file 1471-2148-13-84-S5.doc]

Table S4. DNA site variation and tree statistics for the six datasets used in the phylogenomic analyses presented in this study.

|  | Informative sites (%) | Trees (n) | Tree length | CI | RI | RC |
| --- | --- | --- | --- | --- | --- | --- |
| complete cp genomes | 2.2 | 1 | 14340 | 0.940 | 0.858 | 0.807 |
| coding exons | 1.7 | 1 | 5566 | 0.921 | 0.821 | 0.757 |
| LSC region | 2.7 | 1 | 9922 | 0.935 | 0.842 | 0.787 |
| SSC region | 3.5 | 1 | 2564 | 0.940 | 0.869 | 0.818 |
| IR region | 0.9 | 1 | 888 | 0.970 | 0.931 | 0.902 |
| introns and spaces | 3.2 | 1 | 9202 | 0.932 | 0.834 | 0.778 |

CI, consistency index; HI, homoplasy index; RI, retention index; RC, rescaled consistency index.
