# Supplementary material for: Complete chloroplast genome of the genus Cymbidium: lights into the species identification, phylogenetic implications and population genetic analyses
Source: BMC Evol Biol. 2013 Apr 18;13:84. doi: 10.1186/1471-2148-13-84 (PMC3644226; doi:10.1186/1471-2148-13-84)
Supplement: Additional file 6: Table S5 — Sampled species and voucher specimens of Cymbidium used in this study. [file 1471-2148-13-84-S6.doc]

Table S5. Sampled species and voucher specimens of *Cymbidium* used in this study.

| Taxon | Geographic origin | Voucher | GenBank accession |
| --- | --- | --- | --- |
| *Cymbidium tortisepalum* 1 | Gongshan, Yunnan | HJL091027 | KC876124 |
| *C. tortisepalum* 2 | Baoshan, Yunnan | HJL091035 | KC876128 |
| *C. tortisepalum* 3 | KIB | YJB100601 | KC876125 |
| *C. mannii* 1 | Puer, Yunnan | YJB100602 | KC876129 |
| *C. mannii* 2 | KIB | YJB100603 | KC876126 |
| *C. aloifolium* | Lancang, Yunnan | YJB100604 | KC876122 |
| *C. sinense* | Jingping, Yunnan | YJB100605 | KC876123 |
| *C. tracyanum* | Gengma, Yunnan | YJB100606 | KC876127 |

KIB: Kunming Botanical Garden of the Kunming Institute of Botany
